# Projecting climate change impacts on health: A tutorial integrating the latest climate and demographic scenarios

**DOI:** 10.1097/EE9.0000000000000489

**Published:** 2026-05-19

**Authors:** Marcos Quijal-Zamorano, Pierre Masselot, Antonio Gasparrini, Ana M. Vicedo-Cabrera

**Affiliations:** a Institute of Social and Preventive Medicine (ISPM), University of Bern, Bern, Switzerland; b Center for Climate Change Research (OCCR), University of Bern, Bern, Switzerland; c Environment & Health Modelling (EHM) Lab, Department of Public Health, Environments & Society, London School of Hygiene & Tropical Medicine, London, UK.

**Keywords:** Projections, Health, dlnm, Tutorial, Climate, Temperature

## Abstract

Anthropogenic climate change has led to a widespread and substantial escalation of adverse health impacts, a trend that is expected to amplify in the coming decades under current climate change projections. Thus, it is imperative to generate reliable and robust estimates of climate-sensitive health impacts in future climate change scenarios. Yet, the integration of climate-demographic scenarios and the interpretation of impact projections remain methodologically complex, highlighting the need for more thorough guidance. We present a step-by-step tutorial for conducting health impact projection studies under climate and demographic scenarios. Using heat-related mortality in London as an illustrative example, the tutorial walks the reader through the entire process: from downloading and processing observed and projected climate and demographic data to addressing core methodological challenges, including temporal and spatial alignment, propagating epidemiological and climate uncertainty, and summarizing health impact outputs. To facilitate reproducibility, the tutorial uses an open-access dataset and R code, allowing users to replicate the complete analysis or adapt it to other settings. It serves as a valuable resource for researchers and policymakers by demonstrating how demographics and climate projections jointly influence future health risks, as suggested by the Intergovernmental Panel on Climate Change. By incorporating evolving demographic and climate conditions, it enables more realistic projections of health impacts and provides a stronger foundation for evidence-based adaptation and mitigation strategies.

**What this study adds**

This study provides a practical, step-by-step tutorial for projecting climate-related health impacts that jointly incorporates climate and demographic scenarios, addressing a critical methodological gap in environmental epidemiology. Conducting heat-related mortality projections as an illustrative example, it demonstrated how to process and align climate and population data, propagate uncertainties, and summarize results across time and Global Warming levels. By providing fully reproducible R code and open-access data, the tutorial allows researchers and policymakers to generate robust, transparent, and policy-relevant projections, supporting evidence-based adaptation and mitigation strategies in the context of climate change.

## Introduction

Anthropogenic climate change is driving a rapid increase in global temperatures, a trend expected to continue over the next decades along different trajectories depending on the actions taken to reduce greenhouse gas emissions.^1^ Understanding the potential impact of future warming under different climate change scenarios is a priority in all sectors to anticipate the need for critical infrastructure (i.e., adaptation measures) and boost climate action. Emerging research quantifies future health burdens associated with climate change, particularly temperature-related mortality impacts. Most studies have estimated the projected health impacts by combining historical exposure-response relationships with climate model output under different future emission scenarios.^2–5^ This research has been facilitated by a previous tutorial that provided a step-by-step guide to estimating future health impacts. However, a limitation of this tutorial and most research following its methodology was to consider only changes in climate.^6^

Building on this limitation, it is important to note that climate is only one component of possible scenarios determining future climate-related health burdens. Demographic scenarios, especially under pathways of population aging and declining mortality rates, are also key drivers, given the higher vulnerability of older adults to nonoptimal temperatures.^7–10^ Combining climate and demographic projections into health impact projections nevertheless requires nontrivial methodological steps, including harmonizing temporal and spatial data resolutions, accounting for multiple sources of uncertainty, and interpreting outputs across combined scenarios. Recent initiatives, such as the latest 6th Assessment Report of the Intergovernmental Panel for Climate Change (IPCC),^1^ highlight the need to jointly consider climate and demographic scenarios to obtain more reliable estimates of future health impact trajectories. Only recently have a handful of impact projection studies started to integrate demographic changes, showing that they indeed influence the trends in projected heat-related mortality as much as the warming.^11–14^ However, this methodology can be complex, and there is still no tutorial guiding users and demonstrating how to combine climate and demographic projections within a unified framework.

This work presents an updated tutorial on a more advanced methodological framework that integrates the latest climate and demographic projections with state-of-the-art methods in climate epidemiology. The tutorial is illustrated through a case study using open-access data and is complemented with R code to reproduce the analysis. We show how to account for the contributions of demographics and climate projections, addressing emerging methodological steps that improve the quality of projections but are not covered in the previous tutorial.^6^ We introduce the recent concept of global warming levels (GWL), which has become a widely used approach for presenting climate change projections.^15^ Overall, this advanced framework would support further research on the health impacts of climate change, in line with the latest IPCC recommendations on data and scenarios for impact assessments across sectors.

## Overarching framework and illustrative example

**Figure 1.**
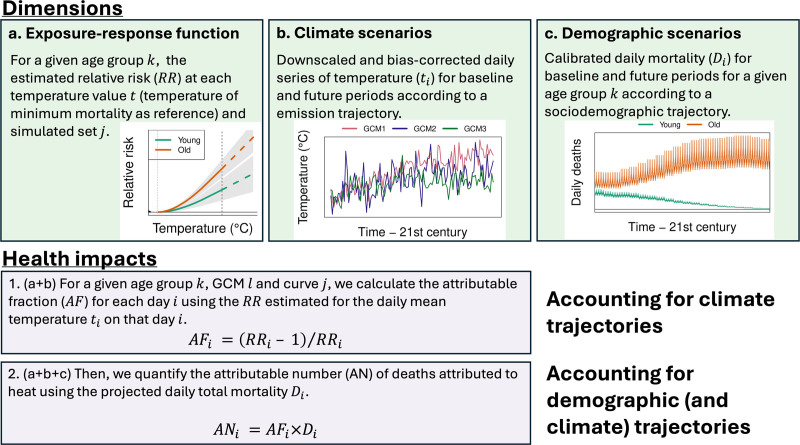
Methodological framework for projecting climate change impacts on health. Summary diagram of the framework presented in this tutorial for projecting the health impacts of climate change. Panels A–C illustrate the processing of epidemiological, climate, and demographic data. Panels 1-2 show how to calculate health impacts accounting for all the dimensions.

Similarly to the previous tutorial,^6^ we illustrate the methodology with a projection study on heat-related mortality in London. Figure 1 summarizes the methodological framework presented in the tutorial. It starts with exposure-response functions (ERF) (section Age-specific exposure-response associations) (i.e., temperature-mortality association) to transform a time series of the projected hazard (i.e., temperature) into projections of relative health outcome increase (so-called attributable fractions [AF]) (section *Climate scenarios*). These impact estimates only account for the climate contribution, with trajectories depending solely on the emission scenario. Then, we calculate the absolute number of the health impact (so-called attributable numbers [AN]) using a baseline incidence of the health outcome (i.e., daily total all-cause mortality) (section *Demographic scenarios*). Until recently, most studies assumed a constant baseline in the future (i.e., the same baseline mortality as in the baseline period).^6^ A key addition in the present tutorial is to relax this assumption by using dynamic projections of baseline incidence in line with the storyline of socioeconomic development. Importantly, this baseline is age-specific and depends on the projected population size, age distribution, and mortality rates. This extension entails using new data, slight modifications to the epidemiological model, and, more importantly, a more complex interpretation of the findings.

In this tutorial, we use an illustrative middle-of-the-road scenario (SSP2-4.5) and calculate heat-related mortality estimates for the full projection period and for specific periods corresponding to a 2°C GWL and the end of the century. Table 1 summarizes the datasets and the corresponding sources used in the illustrative example, all of which are freely available. The following sections provide more details on how to collect and process these datasets, with supplementary information providing additional illustrations of some steps.

**Table 1.** Data sources, characteristics, and preparation steps for the illustrative example of heat-related mortality projections in London

Dataset	Study area	Temporal resolution	Spatial resolution	Time span	Projection scenario	Data preprocessing	Source
1. Temperature and mortality observations	London	Daily	City-level	1990–2012	Not applicable	Age-specific mortality (0–64, 65–74, 75–84, 85 plus) aggregated into <75 and ≥75 age groups	Previous tutorial via GitHub^6^
2. Population observations	London	Annual	City-level	1992–2019	Not applicable	Single-year age-specific populations aggregated into <75 and ≥75 age groups	Eurostat^16^ via eurostat R package^17^
3. Population and survival ratio projections	United Kingdom and Northern Ireland	Quinquennial	National-level	1950–2100	SSP2: Middle-of-the-road	Mortality estimated as population × (1 - survival ratio); age-specific (5-years) mortalities aggregated into <75 and ≥75 age groups	Wittgenstein Centre^18^ via WCDE R package^19^
4. Temperature projections	1°W to 1°E longitude and 51° to 52°N latitude	Daily	0.25 degrees × 0.25 degrees	1950–2100	SSP2-4.5: intermediate GHG emissions	Downloaded for 3 GCMs: BCC-CSM2-MR, MIROC6, IPSL-CM6A-LR; gridded data transformed into a time series for London by averaging cell values within London shapefile boundaries	NEX-GDDP-CMIP6^20^

This table summarizes the key characteristics of the four data sources used for observed and projected climate and demographic information in the illustrative example of heat-related mortality projection in London. The first column describes each dataset’s study area, spatial resolution, temporal resolution, and period covered. The fourth column specifies the selected projection scenarios for demographic and climate projections, but these choices are easily interchangeable with other available options to explore other climate and demographic futures. The data preparation column outlines the technical steps taken to align spatial and temporal resolutions across datasets, an essential step in this type of analysis. The final column provides the original data sources. All data processing steps described in this table are implemented in the R script.

## Age-specific exposure-response associations

A key element in any health impact assessment is the ERF, representing the association between the hazard and the health outcome. When different demographic scenarios are involved, age-specific ERFs become useful. The main reason, as discussed in more detail in section Accounting for climate and demographic scenarios, is that different population development storylines lead to varying population distribution and mortality rates across age groups, which in turn show different ERFs. Therefore, a critical step in this framework is to combine age-specific baseline mortality projections under a given scenario with the corresponding age-specific ERFs.^14^ Different age-group stratifications can be used depending on the research goals and data availability. However, the choice involves a trade-off: age groups should be detailed enough to capture different baseline incidence of the health outcome (e.g., between younger and older populations) but broad enough to ensure sufficient statistical power for estimating age-specific ERFs.

One can obtain ERFs from the literature or estimate them empirically from historically observed data. For example, age-specific ERFs for London could be retrieved from a recent publication.^11,21^ However, for illustrative purposes, we show in this example how to estimate age-specific (<75 and ≥75 years) temperature-mortality associations using the state-of-the-art methods and observed data from London.

### Data collection and preprocessing

We download the age-specific time series of daily observed temperatures and (all-cause) mortality counts from the previous tutorial.^6^ We aggregate these counts into two age groups (<75 and ≥75 years), as shown in the R code to reproduce the tutorial.

### Estimation of age-specific ERFs

For each age group, we apply a quasi-Poisson regression model with distributed lag nonlinear models to estimate the age-specific ERFs (details in Supplementary Text S1; https://links.lww.com/EE/A428).^22^ The model output is a set of estimated coefficients $\theta_{k}$ that define the function $f\left(x_{i};\theta_{k}\right)$, which specifies the association between temperature x at time i and mortality risk for a given age group k. To propagate the uncertainty of epidemiological models, we generate 100 Monte Carlo samples of the coefficients for each group, assuming a multivariate normal distribution for the spline coefficients.^23^ We use a relatively small number of simulations in the tutorial to illustrate the process. However, readers should note that, in applied health impact projection studies, the number of Monte Carlo simulations ranges from 500 to 1,000.^2,3,11^

**Figure 2.**
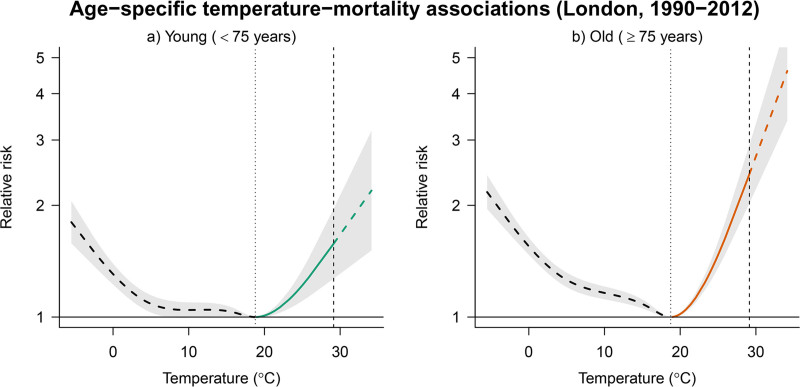
Age-specific (<75 and ≥75 years) temperature-mortality associations in London (1990–2012). Panels A and B show the relative risks of mortality associated with daily temperatures, accumulated across a 21-lag period, with 95% confidence intervals. Heat is defined as the temperature range above the temperature of minimum mortality, indicated by the first vertical dotted line and the right tail of the curve. The dashed segment of the heat tail represents the extrapolation beyond the maximum temperature observed during 1990–2012, marked by the second vertical dashed line.

For each age group (k=1,2) and simulated curve (j=0,1,⋯,100  with j=0 for the estimated coefficients, $j=1,\;\ldots ,\;n_{sim}=100$ for the sampled), the coefficients $\theta_{jk}$ are used to compute the relative risks (RR):


$$RR\left(x_{i},\theta_{jk}\right)=e^{\left[\;f\left(x_{i},\theta_{jk}\right)-f\left(MMT_{k},\theta_{jk}\right)\right]},\;\forall i,j,k,$$

where $MMT_{k}$ is the age-specific minimum mortality temperature.^24^ Figure 2 shows the different risks associated with heat across age groups, with a steeper increase in RR at temperatures above the minimum mortality temperature among older adults compared with younger individuals. Supplementary Figure S1; https://links.lww.com/EE/A428 shows the ERFs obtained with the sampled coefficients, illustrating the uncertainty of the epidemiological model.

As discussed in the previous tutorial, the ERF must be extrapolated beyond the range of the observed temperatures up to the maximum projected value.^6^ For that, we assume a log-linear extrapolation of the risk, following the constraint imposed by the natural spline function of the ERF. For simplicity, in this tutorial, we assume no change in heat-mortality risk over time (i.e., no adaptation), although different ERFs could be used representing various adaptation scenarios.^6,11,25^

## Accounting for climate and demographic scenarios

Climate scenarios are now commonly represented by combinations of socioeconomic scenarios, defined by the shared socioeconomic pathways (SSPs), and radiative forcing scenarios, captured by the representative concentration pathways (RCPs). SSPs outline different futures based on socioeconomic development trajectories (e.g., from sustainable development to fossil fuel-driven growth) and provide quantitative projections of key indicators, including demographic variables such as population change, fertility, mortality, and migration.^18,26^ These projections are derived from the qualitative SSP narratives and support their use as a critical tool for evaluating policy responses and exploring the long-term consequences of climate change.^27,28^ RCPs, in contrast, are direct inputs to general circulation models (GCMs) that describe different trajectories of greenhouse gas concentrations under varying emission levels.^29^

In the latest phase of the Coupled Model Intercomparison Project (CMIP6), five main scenarios spanned a broad range of emissions and warming outcomes and integrate socioeconomic and demographic drivers, emissions, and climate impacts within a coherent cause–effect framework.^1^ In the following subsections, we describe how to select, process, and integrate these scenarios into health impact projections.

### Climate scenarios

For our illustrative example, we first download and arrange temperature projections, which are then transformed into heat-related AFs using the age-specific ERFs.

#### Data collection and preprocessing

For the SSP2-4.5 scenario in London, we use daily temperature projections from three GCMs included in the NEX-GDDP-CMIP6 dataset.^20^ This dataset provides statistically downscaled and bias-corrected temperature projections based on the latest CMIP6 outputs.^11,20,30^ We access the data service and download spatial subsets of the gridded files (1°W–1°E, 51°–52°N) covering London.

#### Processing of the climate data

To obtain city-specific temperature time series, we compute the weighted mean of the grid cell values, using weights proportional to the fraction of each cell within London’s administrative boundaries (Supplementary Figure S2; https://links.lww.com/EE/A428).

**Figure 3.**
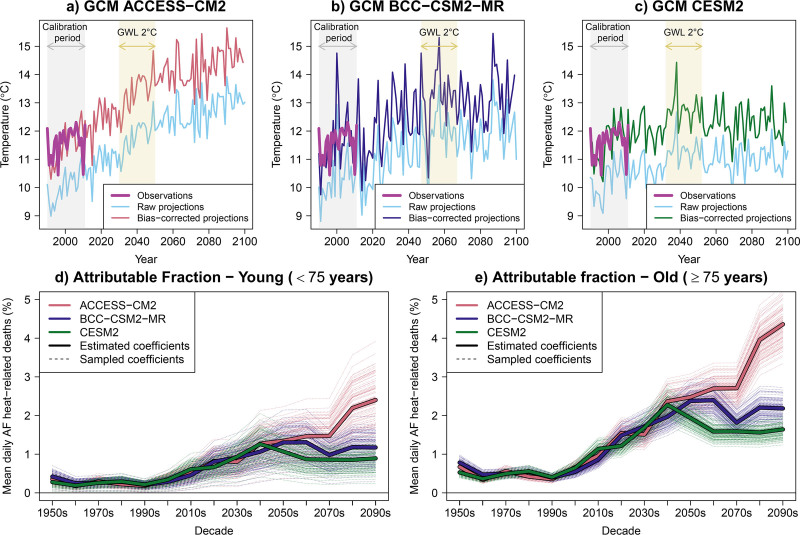
Bias correction of temperature projections and calculation of climate-driven health impacts in London under the SSP2-4.5 climate change scenario (1950–2099). Panels A–C show outputs from the three GCMs used in the illustrative example: ACCESS-CM2, BCC-CSM2-MR, and CESM2. Each panel shows observed annual mean temperature alongside raw and bias-corrected GCM mean annual projections under SSP2-4.5. The shaded areas indicate the 21-year periods centred on the GWL of 2 °C for each model. Panels D and E show the mean of daily AF of heat-related deaths throughout the 21st century, incorporating the uncertainty from both the epidemiological and climate models. Note that yearly or decadal values are shown for visual clarity, but the framework operates at daily resolution.

There can exist systematic differences between data used to estimate ERFs and climate model projections, which can bias health impact estimates.^6^ To alleviate this potential bias, we align temperature projections with the observed data used in the epidemiological models by calibrating GCM outputs using the ISIMIP3BASD method,^31,32^ with the 1990–2011 historical period as the reference.^6^ This ISIMIP3BASD method applies parametric quantile mapping that corrects biases across the full distribution while preserving the long-term trends in each quantile. Extremes are handled via an event-likelihood adjustment that constrains their frequency and magnitude to plausible ranges.^31^ After bias correction, the adjusted climate simulation series align closely with observations during the historical period while preserving the original warming trend (Figure 3A–C).

#### Estimation of the attributable fraction of deaths

Next, for each GCM, we derive daily AF building on the $RR\left(x_{il},\;\theta_{jk}\right)$, which depend on GCM-specific temperatures ($l=1,\;\ldots ,\;n_{gcm}=3)$, calculated as:


$$AF_{ijkl}=\frac{RR\left(x_{il},\;\theta_{jk}\right)-1}{RR\left(x_{il},\;\theta_{jk}\right)},\;\forall i,j,k,l$$ .

In this way, AF captures climate-driven relative health toll across scenarios. To focus on heat-related AFs, we set AFs for temperatures below $MMT_{k}$ equal to zero. Figure 3D,E illustrate these climate-driven health trajectories, showing different AFs across GCMs in addition to the uncertainty from the epidemiological models.

### Demographic scenarios

Next, for the illustrative example, we download and process age-specific mortality projections so that the baseline health outcome, independent of climate, is incorporated into the calculation of heat-related ANs.

#### Data collection and preprocessing

We obtain quinquennial projected population counts and survival ratios, disaggregated by 5-year age groups and sex, for the United Kingdom and Northern Ireland (UK and NI) from 1950 to 2100 under the SSP2 from the Wittgenstein Centre Human Capital Data Explorer.^33^ This platform provides access to the 2023 update of the SSPs as well as historical reconstructions of the global population by age, sex, and education.^18^ An R package is also available for direct access.^19^

#### Processing of the demographic data

The main challenge is that SSP projections are only available at the national level and for multi-year steps, whereas health impact projection studies often focus on city or regional scales and require daily data. Using unadjusted SSPs would therefore misrepresent local demographic patterns. So, raw data needs to be processed in several steps, as described here.

We first derive all-cause mortality projections (quinquennial and by 5-year categories) by multiplying the population by one minus the survival ratio. We then aggregate the 5-year age-specific and sex-specific mortality groups into the two age categories k (<75 and ≥75 years). Note that one could also integrate SSPs scenarios through changes in vulnerability (i.e., by modifying the ERF via factors such as greenness). However, in the present work, SSPs are included only through demographic changes in population at risk (referred to as “demographic scenarios”).

**Figure 4.**
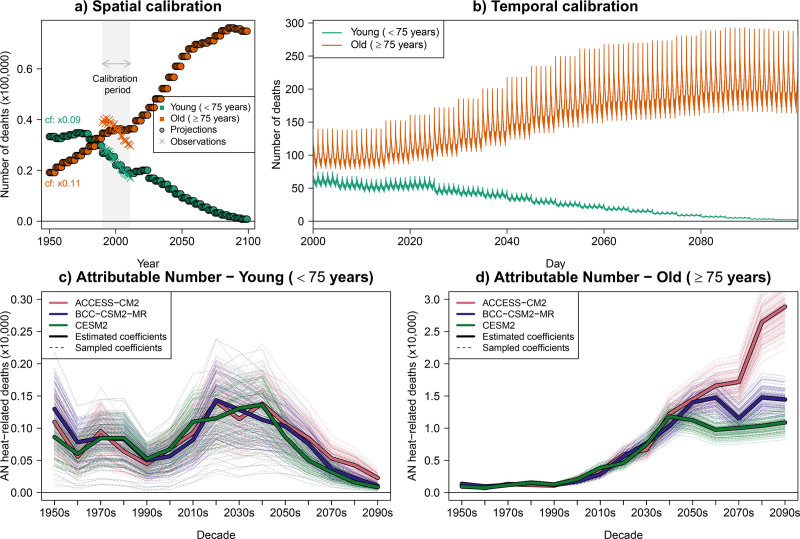
Spatial and temporal calibration of age-specific demographic projections and calculation of demographic- and climate-driven health impacts in London under the SSP2-4.5 climate change scenario (1950–2099). Panel (A) shows spatially calibrated age-specific projections of mortality for younger (<75 years) and older (≥75 years) age groups in London under SSP2, together with the observed annual values used to calibrate the national-level projections (originally provided for the United Kingdom and Northern Ireland). Panel (B) shows the temporally calibrated daily mortality projections (2010–2099), assuming a within-year seasonal pattern during the historical period (1990–2012). Panels (C and D) show the AN of heat-related deaths throughout the 21st century, incorporating the uncertainty from both the epidemiological and climate models. Note that yearly or decadal values are shown for visual clarity, but the framework operates at daily resolution.

We correct the spatial misalignment (national to London) by applying correction factors to the national mortality series. This factor is the ratio of observed (city-level) to projected (national-level) mortality means during the overlapping historical period (1990–2011). The full national projection series is then scaled by this ratio, as shown in Figure 4A. It is to be noted that this approach does not fully capture local trends when correcting, as illustrated by observed older-age mortality in London, which declined during the historical period, whereas the national-level older-age mortality projections remained relatively flat (Figure 4A)—but this approach provides a practical solution to align national projections to city-level historical mean in the absence of finer-scale projections. Interest in obtaining AN rate projections would require applying this same spatial calibration to the national population projections to ensure consistency.

Mortality series display strong seasonality, and ignoring it risks overestimating heat-related mortality and underestimating cold-related mortality in many countries. To account for this when aligning temporal resolutions, we propagate the observed seasonal structure to future daily baseline mortality by distributing 5-year mortality projections across days, assuming the historical seasonal pattern, thereby generating age-specific (k) daily (i) demographic projections $D_{ik}$ (Figure 4B). Specifically, the historical seasonal pattern is obtained by averaging observed mortality counts for each day of the year (Supplementary Figure S3; https://links.lww.com/EE/A428), following the approach described in the previous tutorial and applied in other projection studies.^3,4,6^ Although future mortality seasonality may change under climate and demographic changes, as well as adaptation, including such changes would require substantially more complex mortality forecasting models and is constrained due to limited data available to support SSP-consistent projections of future mortality seasonality. Assuming a fixed historical seasonal pattern fits the historical period well, but may underestimate heat-related mortality projections if seasonal patterns shift with rising temperatures.^4,34^ An alternative is to assume constant mortality within each year, implying identical baseline mortality across seasons. However, in temperate zones with higher winter mortality, this assumption may lead to an overestimation of heat-related mortality during warmer months in the historical period.

#### Estimation of the attributable number of deaths

The AN is then obtained by multiplying the AF by this baseline D of the health outcome:


$$AN_{ijkl}=D_{ik}\times AF_{ijkl},\;\forall i,j,k,l.$$

As shown in Figure 4C, the low baseline mortality in younger age groups drives their heat-related mortality to minimal values by the end of the century. In contrast, the combination of rising temperatures and higher baseline mortality leads to increasing heat-related mortality in older groups, offsetting the decline seen in the younger ones (Figure 4D).

## Aggregating health impact projections outputs

The outputs obtained in previous steps were presented at daily resolution i, by age group k, and for each GCM run l and simulated set of coefficients j, retaining the full uncertainty of climate and epidemiological models. In practice, however, results are usually reported for specific time windows (e.g., decades) and aggregated populations, expressed as point estimates with 95% empirical confidence intervals (CIs) that combine all sources of uncertainty. This section describes the final steps to produce such summary outputs.

Because the analysis is performed at a daily resolution and by age group, results can be flexibly aggregated over time and demographic dimensions. The health impact over a given temporal window T or demographic group G is obtained by summing the corresponding ANs:


$$ANagg_{jl}=\underset{i\in T}{\sum }\;\underset{k\in G}{\sum }\;AN_{ijkl},\;\forall j,l.$$

For the definition of the temporal window of aggregation, the use of GWL has recently gained attention,^15^ as highlighted in the 6th IPCC Assessment Report.^35^ GWLs are specific global temperature rises (e.g., 1.5, 2, and 3 °C) measured relative to the preindustrial temperatures, and they provide a standardized metric to compare risks across scenarios, since many climate variables exhibit consistent geographical patterns at identical warming levels, regardless of the time or pathway by which they are reached.^1^ In practice, we extract the years each GCM exceeds a 2 °C GWL from the Working Group I Atlas GitHub Repository,^11,36^ and define a 21-year window centered on that exceedance year.^11^ Note that in this case, period T is GCM dependent because each GCM reaches a given GWL at different time points, depending on the climate sensitivity of the SSP-RCP scenario used (Figure 3A–C).

Finally, to summarize the uncertainty arising from both the epidemiological and climate, we first take as the point estimate the mean of ANs across GCMs, computed using only the estimated coefficients of the ERF (j=0):


$${\hat{AN}}_{out}=\left(\overset{n_{gcm}}{\underset{l=1}{\sum }}ANagg_{j=0,l}\right)/n_{gcm}.$$

Then, we compute 95% empirical CI as the 2.5th and 97.5th percentiles of the ensemble of ANs obtained from the sampled ERF coefficients (j≠0) and GCMs:


$$CI_{95\;\%\;}\left(AN_{out}\right)=\left[p_{0.025}\left(ANagg_{j\neq 0,l}\right),p_{0.975}\left(ANagg_{j\neq 0,l}\right)\right],$$

where $p_{y}\left(x\right)$ denotes the y-th percentile of the distribution x. Note that the computation of uncertainty can be more complex and computationally intensive in multi-location analyses.^37^

**Figure 5.**
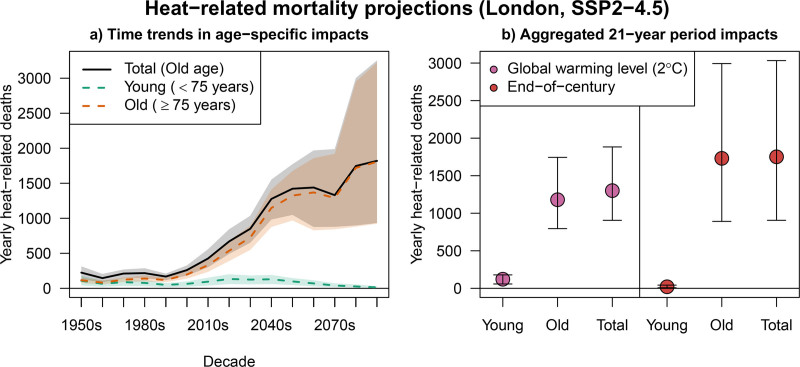
Summary outputs of heat-related mortality projections in London under SSP2-4.5. Panel (A) shows total and age-specific (<75 and ≥75 years) annual heat-related mortality in London for the period 1950–2099, with solid lines indicating point estimates and shaded areas representing 95% confidence intervals. Panel (B) compares projected annual heat-related mortality for two 21-year periods: one corresponding to a global warming level of 2 °C and the other to the end-of-century period (2079–2099). Dots represent point estimates, and error bars indicate 95% confidence intervals.

Figure 5A shows the summary statistics of the projected increase in total and age-specific heat-related mortality in London throughout the 21st century under SSP2-4.5. These outputs summarize the ensemble of health impacts derived from the different climate and epidemiological models shown in Figure 4C, D. Figure 5B compares the health impacts across different periods: yearly ANs at a GWL of 2 °C and for the end of the century (2079–2099).

## Discussion

In this tutorial, we provide an updated step-by-step guide for conducting health impact projection studies considering the most recent climate and demographic data. Through an illustrative example of heat-related mortality projections in London, we guide the reader through the entire process: from downloading and processing observed and projected climate and demographic data to addressing key complexities, including temporal and spatial resolution alignment, uncertainty handling, and combined scenario interpretation. This tutorial enables researchers to conduct tailored health impact projection studies by using the provided R scripts.

Our framework has some limitations that should be mentioned. Although our example assumes no adaptation and uses a limited set of demographic and climate scenarios, it can be extended to more complex scenarios accounting for adaptation (e.g., using different ERFs)^6,11,25^ and diverse socioeconomic and climate projections. While we demonstrated the framework using temperature and mortality data, it is generalizable to a wide range of exposure and outcome variables, as long as data characteristics (e.g., discrete vs. continuous, skewed vs. symmetric) are appropriately accounted for in the epidemiological models and in the calibration of the projections.

In addition, the spatial and temporal coarseness of available mortality projections results in important challenges for calibration to observed data. We described in previous sections the set of assumptions required for this step. Although more sophisticated calibration methods could be applied, our approach (applying correction factors to national projections and assuming constant seasonal patterns) provides a reasonable balance between accounting for these misalignments and keeping the overall process simple. As shown, for example, by population projections downscaled to finer spatial and temporal resolutions,^38,39^ more refined SSP demographic projections could be developed, which may be beneficial for the framework.

Finally, we showed how to account for uncertainty from the epidemiological and climate models. However, demographic uncertainty is not included, as only a single demographic projection is available for each SSP scenario, which prevents the propagation of uncertainty in this component due to the lack of alternative projections or explicit uncertainty characterization.

The presented framework aligns with the latest IPCC approach by combining socioeconomic and climate pathways.^1^ Indeed, previous studies showed the importance of incorporating demographic dynamics, such as an aging population, into health impact projections.^11–14^ Explicit consideration of vulnerable population groups and their evolving demographic trends is essential for accurate health risk assessment under climate change. Thus, by illustrating impacts across socioeconomic scenarios linked to climate mitigation trajectories, health impact projection studies can more effectively inform policymaking and help build more resilient populations to the effects of climate change.

## Conflicts of interest statement

The authors declare that they have no conflicts of interest with regard to the content of this report.

## Supplementary Material
