# Supplementary material for: Projecting climate change impacts on health: A tutorial integrating the latest climate and demographic scenarios
Source: Environ Epidemiol. 2026 May 19;10(3):e489. doi: 10.1097/EE9.0000000000000489 (PMC13189561; doi:10.1097/EE9.0000000000000489)

Supplementary data

Table of Contents

Text S1. Epidemiological models.. 2

Figure S1: Monte Carlo simulations of age-specific (<75 and ⩾75 years) temperature-mortality associations in London (1990-2012)..................................... 3

Figure S2: Construction of the daily temperature projection time series from gridded Global Circulation Models (GCMs). 4

Figure S3: Seasonal pattern of within-year, age-specific mortality in London (1990-2012).. 5

Figure S4: Global warming levels (GWL) periods under the SSP2-4.5 climate change scenario.. 6

Text S1. Epidemiological models.

In section 3, “Age-specific exposure-response associations”, we estimate the association between temperature and mortality for two age groups (<75 and ≥75 years) in London during 1990–2012 using stratified quasi-Poisson regression models combined with distrusted lag non-linear models (DLMNs). The formula used for the two stratified models is:

$$\log(E(mort)) = intercept + S(time, 8\ df\ per\ year) + dow + cb$$

where mort denotes the daily mortality counts for the specific age group, E(mort) is the expected value, S is a natural cubic spline of time with 8 degrees of freedom per year to adjust for the seasonal and long-term trends, dow is a categorical variable controlling for day of the week, and cb is the cross-basis function derived from the DLNM, capturing both the exposure-response and lag-response relationships.[1]

The exposure-response relationship is modelled using a natural cubic spline with three internal knots placed at the 10th, 75th, and 90th percentiles of the observed distribution of daily temperatures. Using the natural cubic spline to model the exposure–response relationship, we ensure a log-linear extrapolation beyond the observed temperature range.[2] The lag-response relationship is modelled using a natural cubic spline with three internal knots equally spaced on the log scale, a maximum lag of 21 days, and an intercept.

We extract the cumulative temperature-mortality association using the reduced model coefficients.[3] Finally, we identify the minimum mortality temperature (i.e., the temperature associated with the lowest estimated risk) which serves as the baseline temperature for the age-specific models: 18.8°C for individuals <75 years and 18.7°C for those ≥75 years.

References

1. Gasparrini A, Armstrong B, Kenward MG. Distributed lag non-linear models. *Stat Med*. 2010;29(21):2224-2234. doi:10.1002/sim.3940

2. Vicedo-Cabrera AM, Sera F, Gasparrini A. Hands-on Tutorial on a Modeling Framework for Projections of Climate Change Impacts on Health. *Epidemiology*. 2019;30(3):321-329. doi:10.1097/EDE.0000000000000982

3. Gasparrini A, Armstrong B. Reducing and meta-analysing estimates from distributed lag non-linear models. *BMC Med Res Methodol*. 2013;13(1):1-10. doi:10.1186/1471-2288-13-1

Figure S1: Monte Carlo samples of age-specific (<75 and ≥75 years) temperature-mortality associations in London (1990-2012).

Panels a-b show the 100 samples from the Monte Carlo simulations representing the relative risk of mortality associated with daily temperatures, accumulated across 21-lag period. These samples are used to propagate epidemiological uncertainty in the temperature-mortality associations. Heat is defined as the temperature range above the temperature of minimum mortality, indicated by the first vertical dotted line and the right tail of the curve. The dashed segment of the heat tail represents the extrapolation beyond the maximum temperature observed during 1990-2012, marked by the second vertical dashed line.

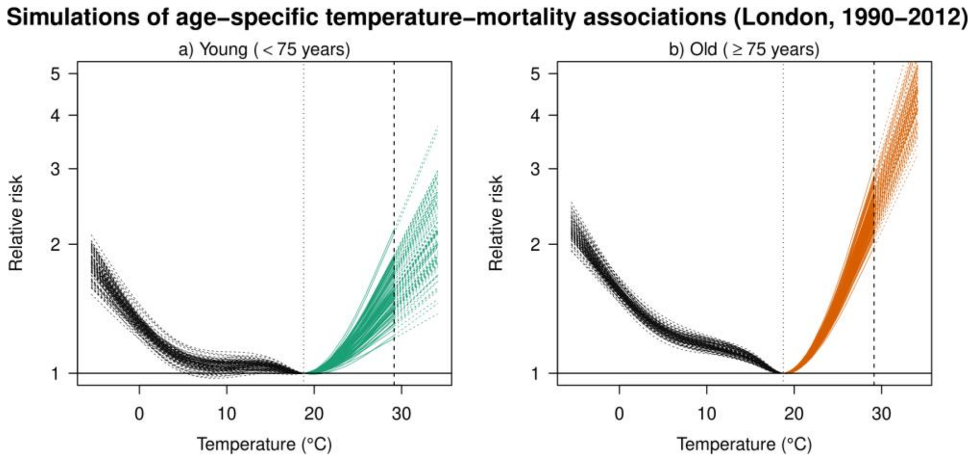

Figure S2: Construction of the daily temperature projection time series from gridded Global Circulation Models (GCMs).

Rows correspond to three selected dates (2073-07-17, 2073-07-18, and 2073-07-19), and columns represent the three GCMs used in the study (ACCESS-CM2, BCC-CSM2-MR, CESM2). Each panel shows the gridded daily mean temperature projections for the area surrounding London. To derive a single representative temperature value for the city (value indicated by the arrow), we compute the weighted mean of the grid cell values, where weights correspond to the proportion of each cell falling within the administrative boundary of London (polygon shown in each panel). This extraction was performed using the exactextractr package in R.[4] The same process can also be applied to other daily gridded observational or reanalysis datasets, such as E-OBS or ERA5-Land,[5,6] to construct observed temperature time series.

References

4. Daniel Baston. exactextractr: Fast Extraction from Raster Datasets using Polygons. 2023, DOI: 10.32614/CRAN.package.exactextractr.

5. Muñoz Sabater J. ERA5-Land hourly data from 1981 to present. *Copernicus Climate Change Service (C3S) Climate Data Store (CDS)* 2019, DOI: 10.24381/cds.e2161bac.

6. Cornes RC, van der Schrier G, van den Besselaar EJM *et al.* An Ensemble Version of the E-OBS Temperature and Precipitation Data Sets. *Journal of Geophysical Research: Atmospheres* 2018;**123**:9391–409.

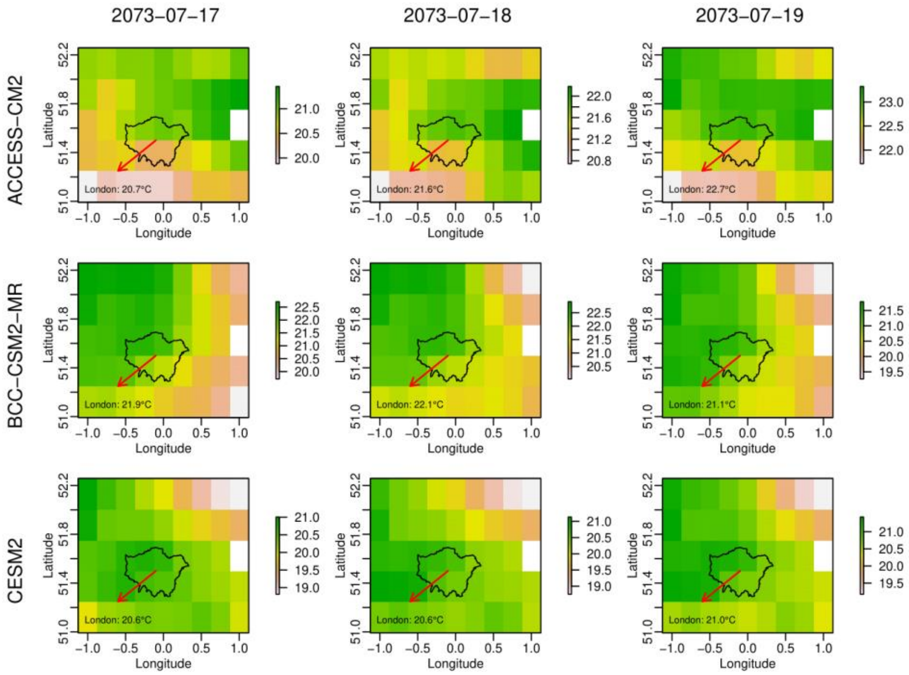

Figure S3: Seasonal pattern of within-year, age-specific mortality in London (1990-2012).

Gray dots represent the percentage of observed annual mortality counts recorded on each day of the year between 1990 and 2011. Blue lines show the within-year seasonal pattern (mean percentage of annual deaths per day of the year in 1990-2012), used to spatially calibrate mortality projections.

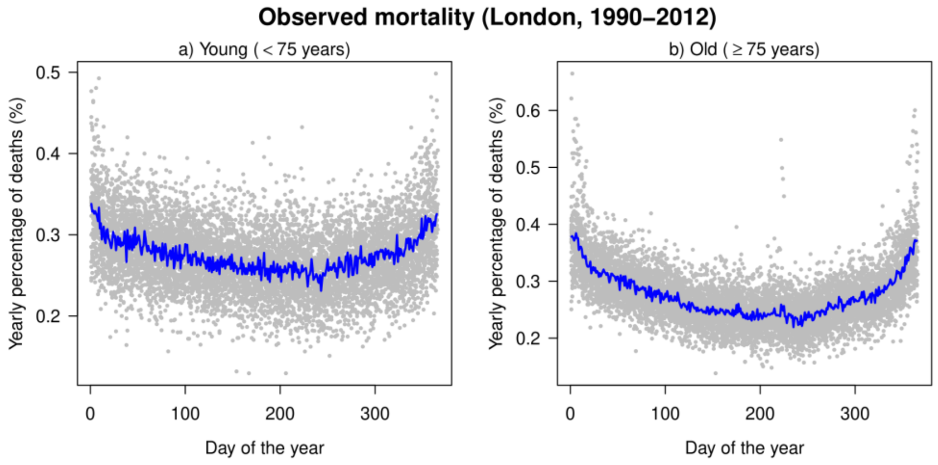

Figure S4: Global warming levels (GWL) periods under the SSP2-4.5 climate change scenario.

Symbols indicate the projected years in which the 1.5°C (square), 2°C (circle), and 3°C (triangle) global warming thresholds are reached according to each of the three selected General Circulation Models (ACCESS-CM2, BCC-CSM2-MR, and CESM2) under SSP2-4.5. Horizontal lines represent the corresponding 21-year periods centred on the year each GWL is reached, as used in the illustrative example.

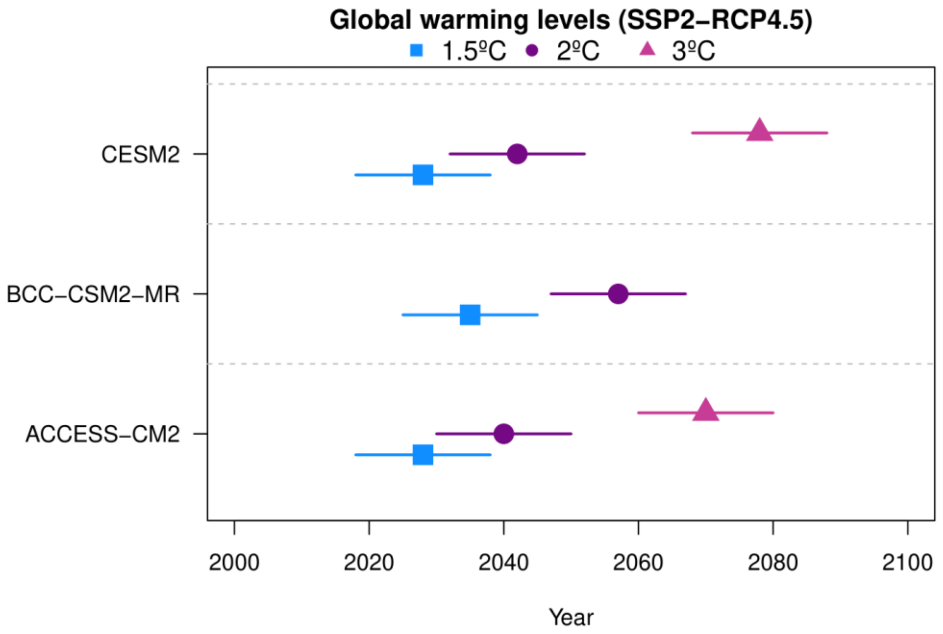

Supplement: Supplementary file 1 [file ee9-10-e489-s001.pdf]
